# Demographic, clinical, and laboratory factors associated with renal parenchymal injury in Iranian children with acute pyelonephritis

**DOI:** 10.1186/s12879-021-06798-x

**Published:** 2021-10-24

**Authors:** Daryoosh Fahimi, Leila Khedmat, Azadeh Afshin, Mohsen Jafari, Zakeyeh Bakouei, Effat Hosseinali Beigi, Mohammad Kajiyazdi, Anahita Izadi, Sayed Yousef Mojtahedi

**Affiliations:** 1 Children’s Hospital Medical Centre, Tehran University of Medical Sciences, Tehran, Iran; 2 Health Management Research Center, Baqiyatallah University of Medical Sciences, Tehran, Iran; 3 Department of Pediatric Nephrology, Bahrami Hospital, Tehran University of Medical Sciences, Tehran, Iran; 4 Department of Pediatric Infection Disease, Tehran University of Medical Sciences, Tehran, Iran; 5 Faculty of Medicine, Tehran University of Medical Sciences, Tehran, Iran; 6 Department of Pediatric Intensive Care Unit, Bahrami Children’s Hospital, Tehran University of Medical Sciences, Tehran, Iran; 7 Department of Pediatric Hematology and Oncology, Tehran University of Medical Sciences, Tehran, Iran

**Keywords:** Acute pyelonephritis, Children, Renal scan, Tc-99m DMSA, Laboratory markers

## Abstract

**Background:**

The association between renal parenchyma changes on dimercaptosuccinic acid (DMSA) scans and demographic, clinical, and laboratory markers was assessed in pediatric patients with acute pyelonephritis.

**Methods:**

A retrospective study of 67 Iranian babies and children aged 1-month to 12-year with APN was conducted between 2012 and 2018. The presence of renal parenchymal involvement (RPI) during APN was determined using technetium-99m DMSA during the first 2 weeks of hospitalization. The association of DMSA results with demographic data, clinical features (hospitalization stay, fever temperature and duration), and laboratory parameters such as pathogen type, and hematological factors (ESR, CRP, BUN, Cr, Hb, and WBC) was evaluated.

**Results:**

92.5% of children with an average age of 43.76 ± 5.2 months were girls. Twenty-four children (35.8%) did not have renal parenchymal injury (RPI), while 26 (38.8%) and 17 (25.4%) patients showed RPI in one and both kidneys, respectively. There was no significant association between RPI and mean ESR, CRP, BUN, and WBC. However, there were significant associations between RPI and higher mean levels of Cr, Hb, and BMI.

**Conclusions:**

Low BMI and Hb levels and increased Cr levels might be indicative of the presence of RPI in children with APN.

## Background

Urinary tract infection (UTI) is one of the most common childhood bacterial diseases worldwide [1–3]. It is more prevalent in girls than boys [4]. Upper UTI could cause renal parenchymal injury (RPI) and subsequent progression to formation of renal parenchymal scars. Moreover, UTI increases the level of stress and anxiety among children and their parents [1, 5, 6]. UTI can involve the upper tract especially kidneys (acute pyelonephritis (APN)) and/or the lower urinary tract including urethra (urethritis) or the bladder (cystitis) [2–4]. However, it is difficult to discriminate them from each other in babies and young children based on clinical symptoms and laboratory results [7]. Finding the proper methodologies for timely diagnosis and treatment is essential because any delay significantly increases the risk of complications such as hypertension, preeclampsia, growth retardation, and progressive kidney scarring and failure later in life [8–10].

Researchers have been looking for affordable and accessible markers with maximum sensitivity and specificity and the least invasive to predict UTI with RPI. The differentiation between upper and lower UTIs, the tracing of abnormalities, and the implementation of appropriate follow-up measures among pediatric patients have been made possible recently [11–13]. Technetium-99m dimercaptosuccinic acid (Tc-99m DMSA) is a standard imaging technique to diagnose RPI [14, 15]. The technique contributes to the definitive assessment of separate kidney function and total radionuclide uptake. A reduction in the radionuclide uptake can be observed in the damaged locations of kidneys. However, if a DMSA scan is normal during a febrile UTI, no scarring will result from that infection [16]. It could also be used to identify the extent of the RPI in the acute phase and on subsequent follow-up [17].

Factors associated with the risk of RPI in children with APN include gender, age, urinary abnormalities (especially vesicoureteral reflux (VUR)), bacterial virulence, recurrence of infection, and bladder dysfunction [18]. Renal parenchymal damage is significantly associated with an increase in levels of laboratory markers of inflammation such as white blood cells (WBCs), erythrocyte sedimentation rate (ESR), and C-reactive protein (CRP) [15, 19]. Accordingly, it seems that the comparison of demographic and laboratory markers with Tc-99m DMSA scans can contribute to an understanding of the risk factors that are predictive of the presence of RPI in children with APN and could therefore serve as early prognostic parameters. Therefore, this 10-year retrospective study aimed to explore the relationships between demographic, clinical, and laboratory indicators and renal DMSA scans of Iranian babies and children with febrile UTI.

## Methods

### Study design and subjects

This study was retrospectively conducted based on the data census of all pediatric patients with APN. All the patients were admitted to Bahrami Hospital (Tehran, Iran), affiliated with Tehran University of Medical Sciences (TUMS), between March 2012 and March 2018. A total of 67 children with APN were evaluated. Verbal and written informed consent was obtained from their parents using phone contact and face-to-face interview methods. Each child had a single code number to maintain the confidentiality of participants' data. The research procedure was entirely consistent with the Human Ethics Committee of the TUMS.

### Inclusion and exclusion criteria

All 1-month to 12-year-old patients with APN admitted to Bahrami Hospital were included in the study. The criteria for diagnosis of pyelonephritis included axillary temperatures of over 38.5 °C, poor general condition as indicated by presence of abdominal and flank pains, vomiting, and agitation [20], and positive urine culture (PUC). A PUC was defined as presence of colony count in midstream urine sample more than or equal to 10^5^ CFU/mL of a single pathogen, or ≥ 10^4^ CFU/mL in urine sample obtained by catheter method, or ≥ 10^3^ Gram-positive bacteria or any number of gram-negative bacteria in urine obtained by suprapubic aspiration (SPA) [21]. Suprapubic aspiration was carried out in babies and children aged 1 month–1.5 years of age, catheterization in 1.5–4 years of age, and clean catch urine collection (Quick-Wee) in 4.5–12 years old [22]. Children with all three criteria were included in the study, while patients with a history of urinary reflux, VUR, urinary tract surgery, urinary tract abnormality, or previous renal scar (presence of photopenic areas with shrinkage and thinning of the renal cortex on DMSA scan), and any pre-existing malformations were excluded.

### Data collection

The medical information of the patients was collected from archived electronic files available from March 2012 to March 2018. The keyword “pyelonephritis” was first searched to prepare a list of patients. After ensuring the accuracy of pyelonephritis diagnosis, the necessary information was extracted by referring to the history, disease course, and summary of patients' files. The RPI severity during acute febrile UTI using the Tc-99m DMSA was that determined at the first 2 weeks of hospitalization. If the hospital records contained the necessary information, the patients' clinic records were studied to extract their DMSA scan related-information. If the DMSA scan data was ambiguous or insufficient, the patient's parents and specialists were contacted. This DMSA scan information was divided into three groups: (I) patients with normal kidney scans or without RPI (n = 24), (II) patients with RPI in one kidney (n = 26), and (III) patients with abnormal scans in both kidneys (n = 17). In this study, the association of severity of involvement of each of the left and right kidneys in groups II and III with body mass index (BMI), glomerular filtration rate (GFR), and other laboratory factors (ESR > 20 mm/h, CRP > 10 mg/L, leukocytosis (WBCs > 11 × 10^9^/L), and anemia [hemoglobin (Hb) < 10.5 g/dL)] was separately determined [19, 22, 23]. The Kirby–Bauer disk diffusion method was used to assess the antibiotic susceptibility of the bacterial isolates [24].

All the information on eligible patients was imported to pre-prepared questionnaires. The data collected included age, gender, height, weight, BMI, fever degree, duration of fever before and after treatment, duration of hospital stay (HS), and results of urine culture, blood urea nitrogen (BUN), serum creatinine (SCr), Hb, WBCs, CRP, ESR, and estimated GFR (eGFR). The kidney function level at the time of admission was assessed by determination of the eGFR (mL/min/1.73 m^2^) using the following equation [25]: $eGFR=\frac{0.413\times \mathrm{Height }(\mathrm{cm})}{\mathrm{SCr }(\mathrm{mg}/\mathrm{dL})}$

As the level of SCr in children is dependent on their age and gender, this biochemical marker was normalized with the median SCr value at the corresponding age and gender. The body height of children was measured using a stadiometer with an accuracy of 0.1 cm. An infantometer was used to measure the recumbent length of young children (< 24 months old). The body weight was measured by wearing light clothes and no shoes or socks using a standard electronic balance with an accuracy of 0.01 g. The BMI was determined by dividing the weight (in Kg) divided by the square of height (in m).

### Data analysis

The data at a significant level of p < 0.05 were analyzed using the SPSS software package version 21.0 (SPSS Inc., Chicago, IL, USA). The descriptive data are represented as frequencies, percentages, and means ± standard deviations. The significance of differences between means and frequencies was assessed using independent t-test (continuous variables) and Chi-square (χ^2^, categorical variables). Multiple means were compared using analysis of variance (ANOVA). A multiple logistic regression analysis was also carried out to assess the independence of association of demographic, clinical and laboratory factors with DMSA scan changes. Pearson’s coefficient was considered to find any significant correlation between tested variables.

## Results

Sixty-seven infants and children, 5 boys (7.5%) and 62 girls (92.5%) were included in the study. Overall, 43 (64.2%) had renal parenchymal involvement, RPI, on DMSA scan while 24 (35.8%) did not. Among those with RPI (n = 43), it was unilateral in 26 children (60.5%) and bilateral in 17 (39.5). Among those with unilateral RPI (n = 26), the left kidney was involved in in all 5 boys, and in only 28 (45.2%) of the 62 girls (p = 0.049), while right kidney involvement was present in 1/5 (20.0%) versus 26/62 (41.9%; p = 0.647).

The differences in demographic, clinical and laboratory indices between children with versus without RPI, children with involvement of left versus right versus both kidneys, and those with unilateral versus bilateral involvement are set out in Tables 1 and 2. Table 1 features the differences in means plus/minus standard deviations and Table 2 the differences in frequencies and proportions. The only significant differences were in BMI, and serum creatinine and hemoglobin levels (Tables 1 and 2). Children with RPI had a significantly lower mean BMI (p = 0.045) and higher number with abnormal BNI (p = 0.038), a higher mean serum creatinine level (p = 0.034) and higher number with abnormal levels (p = 0.042), and lower hemoglobin level (p = 0.048) and higher number with anemia (hemoglobin level < 10.5 g/dL [22, 23]; p = 0.035). Those with bilateral RPI similarly had a significantly lower mean BMI (p = 0.039) and higher number with abnormal BNI (p = 0.029), a higher mean serum creatinine level (p = 0.044) and higher number with abnormal levels (p = 0.045), and lower hemoglobin level (p = 0.045) and higher number with anemia (hemoglobin level < 10.5 g/dL [22, 23]; p = 0.040).

**Table 1:** Mean of demographic, clinical, and laboratory indices in relation to presence and pattern of abnormalities on DMSA scan

Demographic/Clinical factor^a^	DMSA scans^b^		p-value	DMSA scans^b^			p-value	DMSA scans^b^		p-value
	RPI (Abnormal, n = 43)	No RPI (Normal, n = 24)		RPI in LKCs (n = 14)	RPI in RKCs (n = 12)	RPI in both (n = 17)		Unilateral (n = 26) RPI	Bilateral (n = 17) RPI	
Age (month)	46.56 ± 42.58	38.75 ± 33.61	0.477	46.10 ± 32.31	45.97 ± 25.69	47.52 ± 18.51	0.821	46.04 ± 29.52	47.52 ± 18.51	0.852
BMI (kg/m^2^)	16.06 ± 1.99^b^	17.37 ± 3.24^a^	0.045*	15.90 ± 2.74^a^	16.47 ± 1.61^a^	14.08 ± 1.74^b^	0.041*	16.17 ± 1.70^a^	14.08 ± 1.74^b^	0.039*
Fever (°C)	38.85 ± 0.51	38.86 ± 0.43	0.972	38.93 ± 0.61	38.86 ± 0.49	38.91 ± 0.42	0.905	38.89 ± 0.43	38.91 ± 0.42	0.916
HS (day)	4.95 ± 2.80	4.50 ± 2.43	0.509	5.12 ± 1.78	4.81 ± 2.09	5.05 ± 3.20	0.532	5.52 ± 2.42	5.05 ± 3.20	0.608
ESR (mm/h)	49.55 ± 29.54	47.84 ± 24.11	0.728	41.65 ± 16.58	44.09 ± 21.36	38.95 ± 24.60	0.053	42.77 ± 18.53	38.95 ± 24.61	0.062
CRP (mg/L)	56.90 ± 37.59	59.45 ± 33.28	0.784	58.54 ± 32.14	56.36 ± 34.21	59.40 ± 41.70	0.755	57.53 ± 35.79	59.40 ± 41.73	0.689
WBC (× 10^9^/L)	14.21 ± 5.40	13.35 ± 5.18	0.528	13.51 ± 4.92	13.29 ± 5.23	14.75 ± 4.50	0.092	13.40 ± 5.92	14.75 ± 04.50	0.796
Hb (g/dL)	10.77 ± 1.26	11.21 ± 1.08	0.161	10.72 ± 1.05	10.81 ± 1.07	10.83 ± 1.50	0.212	10.76 ± 1.35	10.83 ± 1.50	0.405
BUN (mg/dL)	12.20 ± 5.21	11.37 ± 3.97	0.465	12.86 ± 5.84	11.77 ± 6.02	12.79 ± 5.35	0.422	12.35 ± 5.32	12.79 ± 5.35	0.523
SCr (mg/dL)	0.60 ± 0.16^a^	0.54 ± 0.09^b^	0.034*	0.63 ± 0.03^a^	0.58 ± 0.02^b^	0.55 ± 0.15^b^	0.047*	0.61 ± 0.09	0.55 ± 0.15	0.044*
eGFR (mL/min/1.73 m^2^)	65.87 ± 16.25	66.72 ± 14.76	0.829	66.67 ± 9.75	64.40 ± 11.01	63.38 ± 14.74	0.836	65.60 ± 15.33	63.38 ± 14.74	0.687
Hb (g/dL)	11.1 ± 0.9^a^	11.8 ± 1.2^b^	0.048*	10.4 ± 0.6^a^	10.9 ± 0.8^b^	11.2 ± 0.8^b^	0.049*	10.6 ± 0.7^a^	11.2 ± 0.8^b^	0.045*

* p-values less than 0.05 are statistically significant

^a^*BMI* body mass index, *HS* hospitalization stay, *ESR* erythrocyte sedimentation rate, *CRP* C-reactive protein, *WBC* white blood cell, *Hb* hemoglobin, *BUN* blood urea nitrogen, *SCr* serum creatinine, *eGFR* estimated glomerular filtration rate

^b^*RPI* renal parenchymal injury, *LKCs* left kidney changes, *RKCs* right kidney changes

**Table 2:** Relationship between the frequency of abnormal clinical/laboratory indices and presence versus absence of RPI, and pattern of RPI (left versus right versus both kidneys, and unilateral versus bilateral

Demographic/clinical factor^a^	DMSA scans^b^		p-value	DMSA scans^b^			p-value	DMSA scans^b^		p-value
	RPI (Abnormal, n = 43)	No RPI (Normal, n = 24)		RPI in LKCs (n = 14)	RPI in RKCs (n = 12)	RPI in both (n = 17)		Unilateral (n = 26) RPI	Bilateral (n = 17) RPI	
BMI (kg/m^2^)	24 (55.8)	7 (29.2)	0.038*	6 (42.8)	6 (50)	12 (70.6)	0.033*	12 (46.1)	12 (70.6)	0.029*
Fever duration (BT)	12 (27.9)	5 (25)	0.051	4 (28.6)	3 (25)	5 (29.4)	0.662	7 (26.9)	5 (29.4)	0.711
Fever duration (AT)	3 (7)	1 (4.1)	0.754	1 (7.1)	1 (8.3)	1 (5.9)	0.546	2 (7.7)	1 (5.9)	0.684
ESR (mm/h)	7 (16.3)	3 (12.5)	0.125	2 (14.3)	2 (16.7)	3 (17.6)	0.607	4 (15.4)	3 (17.6)	0.655
CRP (mg/L)	2 (4.7)	1 (4.2)	0.711	1 (7.14)	0 (0)	1 (5.9)	0.094	1 (3.8)	1 (5.9)	0.102
WBC (× 10^9^/L)	7 (16.3)	4 (16.7)	0.803	2 (14.3)	2 (16.7)	3 (17.6)	0.607	4 (15.4)	3 (17.6)	0. 655
Hb (g/dL)	3 (7)	2 (8.3)	0.072	1 (7.1)	1 (8.3)	1 (5.9)	0.546	2 (7.7)	1 (5.9)	0.684
BUN (mg/dL)	6 (14)	4 (16.7)	0.314	2 (14.3)	2 (16.7)	2 (11.8)	0.056	4 (15.4)	2 (11.8)	0.052
SCr (mg/dL)	10 (23.3)	3 (12.5)	0.042*	2 (14.3)	4 (33.3)	4 (23.5)	0.039*	8 (30.7)	4 (23.5)	0.045*
eGFR (mL/min/1.73 m^2^)	4 (9.3)	2 (8.3)	0.772	1 (7.1)	1 (8.3)	1 (5.9)	0.546	2 (7.7)	1 (5.9)	0.684
Anemia (Hb < 10.5 g/dL, %)	11 (25.6)	3 (12)	0.035*	6 (42.8)	2 (16.6)	3 (17.6)	0.036*	8 (30.8)	3 (17.6)	0.040*

Frequencies are expressed as number (percentage)

* p-values less than 0.05 are statistically significant

^a^*BMI* body mass index, *BT/AT* before/after treatment, *ESR* erythrocyte sedimentation rate, *CRP* C-reactive protein, *WBC* white blood cell, *Hb* hemoglobin, *BUN* blood urea nitrogen, *SCr* serum creatinine, *eGFR* estimated glomerular filtration rate

^b^*RPI* renal parenchymal injury, *LKCs* left kidney changes, *RKCs* right kidney changes

Table 3 shows that the order of effectiveness of predictors associated with the presence of abnormalities on renal scan changes among the children with APN was Hb (anemia) > SCr > BMI. The correlation results of these parameters revealed that the strongest association was with BMI and SCr levels (p < 0.05; r = 0.894).

**Table 3:** Results of multiple logistic regression for the association between demographic/clinical presence of RPI on DMSA scans

Variables	Beta	Odds ratio	95% CI	p-value
BMI	0.314	1.368	0.932–1.614	0.0411
Hb	− 0.693	0.500	0.276–0.805	0.0193
SCr	3.689	40.004	0.598–2579.1	0.0367

Dependent variable: DMSA renal scan changes (positive: 1, negative: 0)

The patients had a PUC with six pathogen types. As expected, *Escherichia coli* was the most frequent pathogen and was isolated in 89.4% urine samples. The other bacterial species were Gram-negative bacilli (n = 2; 3.03%), *Klebsiella pneumoniae* (n = 2; 3.03%), *Acinetobacter* (n = 1; 1.51%), *Enterococcus* (n = 1; 1.51%), and Group B *Streptococcus* (n = 1; 1.51%). There was no significant association between LKCs and bacterial species. There were three girls with recurrent UTI (4.5%), and *E. coli* was the isolate from these children. The antibiotic susceptibility of the *E. coli* isolates showed the highest prevalence of resistance to ampicillin (87.2%) and cotrimoxazole (83.2%), while the highest prevalence of sensitivity was to gentamicin (75.1%) and imipenem (72.9%).

## Discussion

Our findings showed that both the presence (RPI present versus absent) and pattern (unilateral versus bilateral RPI, and RPI in left versus right kidney) of RPI as diagnosed using DMSA scans had significant associations with only three factors including BMI, SCr, and hemoglobin level.

Obesity is associated with an increased risk of febrile UTI and APN [26–29]. The high BMI in obese children can negatively affect the function of immune cells through the generation of chronic low-grade systemic inflammation, changes in the complex interactions of adipokines, immune cells, and cellular metabolism, as well as epigenetic changes. The dysregulation of immune responses disturbs the normal balance of sympathetic and parasympathetic activity to control voiding and urine storage and leads to UTI or APN [26]. 60–65% of patients with febrile UTIs may have APN [30]. We however found that children with LKCs and bilateral RPI have lower BMI values than those with normal DMSA scans. Thus, low BMI could be also a significant risk factor for UTI and development of severe infection with RPI in children in developing countries (such as Iran), probably reflecting malnutrition and poor hygiene standards. The weak immunity among undernourished persons has been attributed to the depleted leucocyte, lymphocyte, and T-cell counts, increased CD4/CD8 ratios, and decreased CD2/CD19 ratios [31, 32]. However, optimal antimicrobial therapy may improve the BMI effect on the gut microbiota [27, 33], and the linkage between changed gut microbiota and human metabolism [34], as well as bone growth and development [35].

There was a significant association between the presence of renal parenchyma changes and increased SCr. Similarly, Ataei et al. [36] reported a significant correlation between renal parenchyma changes and increased SCr, but Amaral et al. [37] did not report any. This discrepancy may be due to the unbalanced alterations in muscle mass and tubular secretion and their effects on the SCr levels [38]. Breinbjerg et al. [39] also found that even non-*E. coli* infections can lead to an elevated SCr in children after their first ANP. In addition, Megged [40] found that SCr was the only independent risk factor for bacteremia among children with UTI. This association could be because hypotension from bacteremia decreases blood flow to the kidneys, resulting in increased SCr levels [41]. The oxidative stress induced by reactive oxygen species during infection along with a reduction in the concentration of antioxidant enzymes (e.g., superoxide dismutase (SOD), catalase (CAT), and glutathione peroxidase (GPx)) may also be an important factor in the pathogenesis of kidney damage and elevation of SCr levels [41–43].

Compared with SCr and BMI, the presence of anemia was more indicative of the presence and severity of APN. The low Hb levels in children with APN may be due to activation of the renin–angiotensin–aldosterone system, leading to enhanced tubular reabsorption of water and consequent dilutional anemia [44]. In addition, Viana et al. [45] have pointed out that a number of acute bacterial/viral infections can also lead to anemia through mild idiopathic hemolysis, marrow inhibition, and iron deficiency.

In this study, no significant associations were identified between the presence of RPI and the other clinic-laboratory parameters. Ataei et al. [36] did not also report any significant correlations between the presence of abnormalities on Tc-99m DMSA scan and hematological parameters like WBC, ESR, and CRP nor did. Jaksic et al. [30] also report any significant association with gender, age, temperature, ESR, and CRP.

Patients with more severe PI had a longer duration of fever, both before and after the commencement of treatment. This could be because the formation of renal scar is more a reflection of inflammatory tubular damage than bacterial growth in the kidneys. Fever is a result of the local inflammatory response to circulating immune cells and the production of different acute-phase proteins. Therefore, further proliferation and renal scar can be stimulated at higher fever degrees [46]. *E. coli* was the most common bacterial isolate from children with UTI. This pathogen and other Enterobacteriaceae, because of the biosynthesis of hydrolytic enzymes like extended-spectrum β-lactamase, have a high ability to acquire resistance against a wide spectrum of antibiotics. Therefore, it is necessary to choose an efficient antimicrobial agent to treat pediatric UTIs [47].

## Conclusions

We conclude that a lower BMI, higher SCr, and anemia are significantly associated with the presence of RPI in children with febrile UTI. Evaluating these demographic and laboratory features might be helpful to identify pediatric patients with APN who require a close follow-up of renal function.

## Data Availability

The datasets used and/or analyzed during the current study are available from the corresponding author on reasonable request.

## Abbreviations

APN: Acute pyelonephritis
BMI: Body mass index
BUN: Blood urea nitrogen
CRP: C-reactive protein
ESR: Erythrocyte sedimentation rate
(e)GFR: (Estimated) glomerular filtration rate
Hb: Hemoglobin
HS: Hospitalization stay
(R)PI: (Renal) parenchymal involvement
LKCs: Left kidney changes
PUC: Positive urine culture
RKCs: Right kidney changes
SCr: Serum creatinine
Tc-99m DMSA: Technetium-99m dimercaptosuccinic acid
UTI: Urinary tract infection
VUR: Vesicoureteral reflux
WBCs: White blood cells
